# Successful Treatment of Central Nervous System Post-Transplant Lymphoproliferative Disease With a Reduced Dose of High-Dose Methotrexate

**DOI:** 10.7759/cureus.32567

**Published:** 2022-12-15

**Authors:** Linda Albusoul, Ahmad Abu-Hashyeh, Vijayalakshmi Donthireddy

**Affiliations:** 1 Internal Medicine, Henry Ford Health System, Detroit, USA; 2 Hematology and Oncology, Henry Ford Health System, Detroit, USA

**Keywords:** central nervous system post-transplant lymphoproliferative disease, methotrexate, post-transplant lymphoproliferative disease, high-dose methotrexate, kidney transplant

## Abstract

Post-transplant lymphoproliferative disease (PTLD) is a complication of solid organ and hematopoietic stem cell transplantation that occurs as a result of immunosuppression. PTLD isolated to the central nervous system (CNS) is a rare disease and it presents with nonspecific signs and symptoms. Optimal therapy guidelines have not yet been established for CNS PTLD. Here, we report a case of successful treatment of CNS PTLD in an adult female following two subsequent kidney transplants. Initial management was with immunosuppression reduction and a trial of rituximab. There were concerns regarding using methotrexate (MTX) given the patient’s fragile transplant status. Magnetic resonance imaging of the brain following four cycles of rituximab revealed the progression of the disease. Subsequently, high-dose MTX (HD-MTX) was considered within the constraints of potential kidney toxicities given her transplant status and chronic kidney disease. Potential toxicities from other therapies, such as brain radiation, also factored into the final decision. The patient was treated with one cycle of a combination of rituximab and HD-MTX 1 g/m^2^. The patient tolerated HD-MTX and did not have evidence of renal toxicity in laboratory studies. Following that, she was started on a reduced dose of HD-MTX at 2 g/m^2^ every two weeks instead of the higher MTX dose range of 3.5 to 8 g/m^2^, which was a shared decision with the patient and nephrology after weighing the risk of kidney dysfunction with the possibility of a less than optimal response with regards to her lymphoma. She was followed with a magnetic resonance imaging of the brain, which demonstrated a complete response after four cycles. Further consolidation treatments with HD-MTX 2 g/m^2^ every four weeks were administered to complete one year of treatment. Following the completion of chemotherapy, the patient was able to achieve and maintain a complete response without affecting her kidney function. She continues to do well one year following treatment. This case highlights the significance of tailoring therapy to each individual based on their comorbidities and clinical response, as well as the possible merit in exploring the use of a reduced dose of HD-MTX in the treatment of CNS PTLD in patients at high risk for renal toxicity.

## Introduction

Post-transplant lymphoproliferative disease (PTLD) is a heterogeneous group of lymphoid disorders that ranges from indolent polyclonal proliferation to aggressive lymphomas, which complicates solid and hematopoietic stem cell transplantation in the setting of immunosuppression [1]. Lymphoma isolated to the central nervous system (CNS) is a rare occurrence. In a retrospective study that included 5773 kidney recipients, 90 cases of PTLD were identified and only about 7% of these cases had CNS disease [2]. Clinical presentation can be highly variable between patients and may constitute any combination of neurological complaints, including but not limited to confusion, memory impairment, mood changes, cranial nerve involvement, and ataxia [2]. CNS PTLD can pose a diagnostic challenge; in one case series, the mean time between initial symptoms and the time of diagnosis was 3.5 months [3]. Additionally, another case series reported a 36-56-month interval to diagnosis [4]. Delays in diagnosis can be attributed to the rarity of this condition, nonspecific manifestations, as well as frequent co-morbid conditions in this patient population.

The fifth edition of the World Health Organization's classification of hematolymphoid tumors introduced significant changes to the classification of immunodeficiency-associated lymphoproliferative disorders [5]. Previously, PTLD was classified into four major subtypes, which included early hyperplastic lesions, monomorphic, polymorphic, and classical Hodgkin lymphoma-like PTLD [6]. Most recent classifications focused on histologic and pathologic features as well as the causal association of specific lesions instead of grouping disorders based on the causative disease [5]. PTLD condition is often associated with Epstein-Barr virus (EBV) infection. However, this does not always correlate with positive cerebrospinal fluid or serum titers as was the case with our patient [7]. EBV plays a crucial role in the development of PTLD in multiple ways through interaction with B lymphocytes and activation of transcription factors [8]. EBV infection is implicated in most early cases of PTLD, and it becomes less frequent with the later presentation. On the other hand, this does not seem to hold true in CNS PTLD, which may indicate the presence of other pathways for lymphoma development that are still not well understood [8].

The optimal therapy options have not yet been established for CNS PTLD. Treatment options for CNS lymphoma include the trial of immunosuppression reduction and the use of rituximab as monotherapy or in combination with other chemotherapy. Additionally, whole-brain radiation can be used [6].

## Case presentation

**Figure 1:**
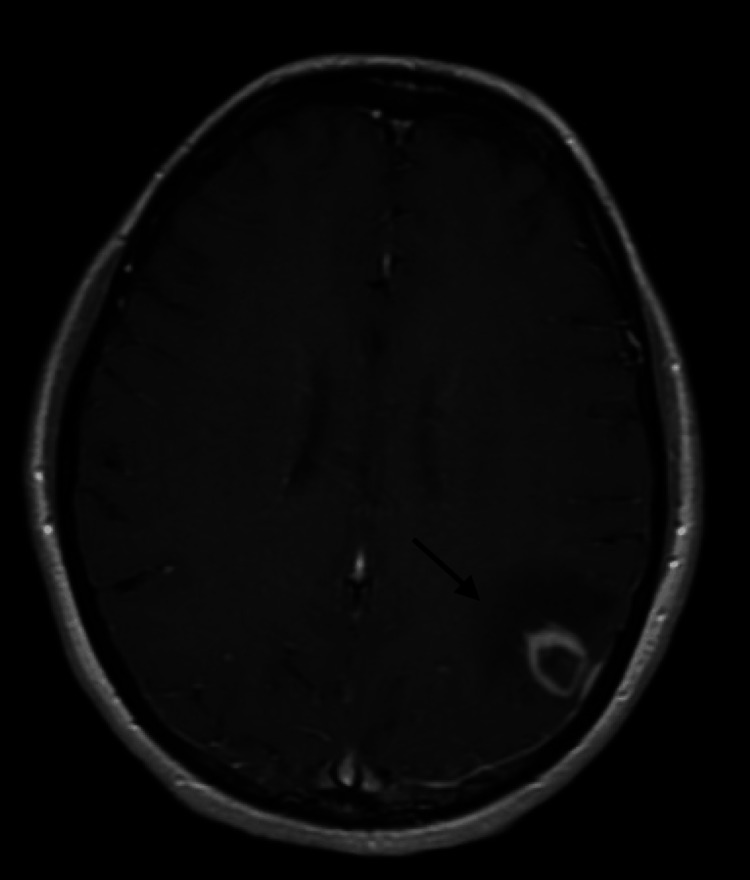
MRI of the brain showing heterogeneously enhancing and centrally non-enhancing lesion within the left parietal lobe. MRI: magnetic resonance imaging

A 47-year-old female patient with a past medical history notable for immunoglobulin A nephropathy ultimately requiring treatment with two kidney transplants, and chronic kidney disease stage 3 who presented with new onset generalized tonic-clonic seizures. Her first kidney transplant was 16 years prior, it was complicated by transplant failure after eight years. She underwent a successful second transplant three years prior to her current presentation. On presentation to the emergency department, she was afebrile and hemodynamically stable. The neurological exam was unremarkable. Laboratory studies, including complete blood count and complete metabolic profile, were unremarkable. Computed tomography of the head and magnetic resonance imaging (MRI) of the brain showed a 20.6 x 19.1 x 17.8 mm left parietal ring-enhancing lesion concerning for malignancy versus infection (Figure 1). Lumbar puncture was negative for infection. Cerebrospinal fluid cytology demonstrated lymphocytic pleocytosis with no definitive evidence of B lymphoid neoplasia.

**Figure 2:**
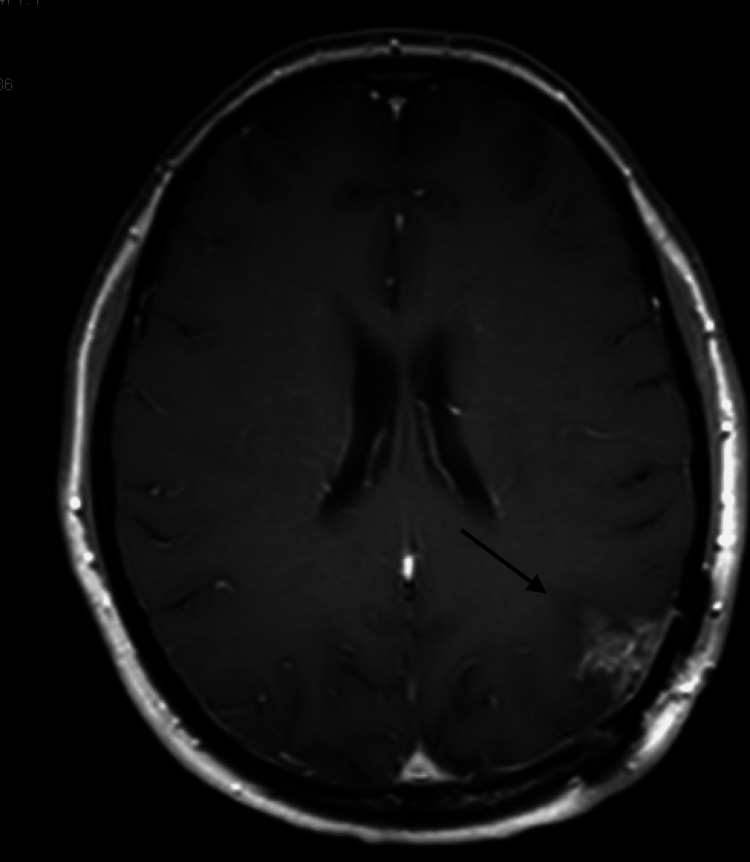
MRI of the brain showing interval development of poorly marginated heterogeneous enhancement of the resection cavity. MRI: magnetic resonance imaging

**Figure 3:**
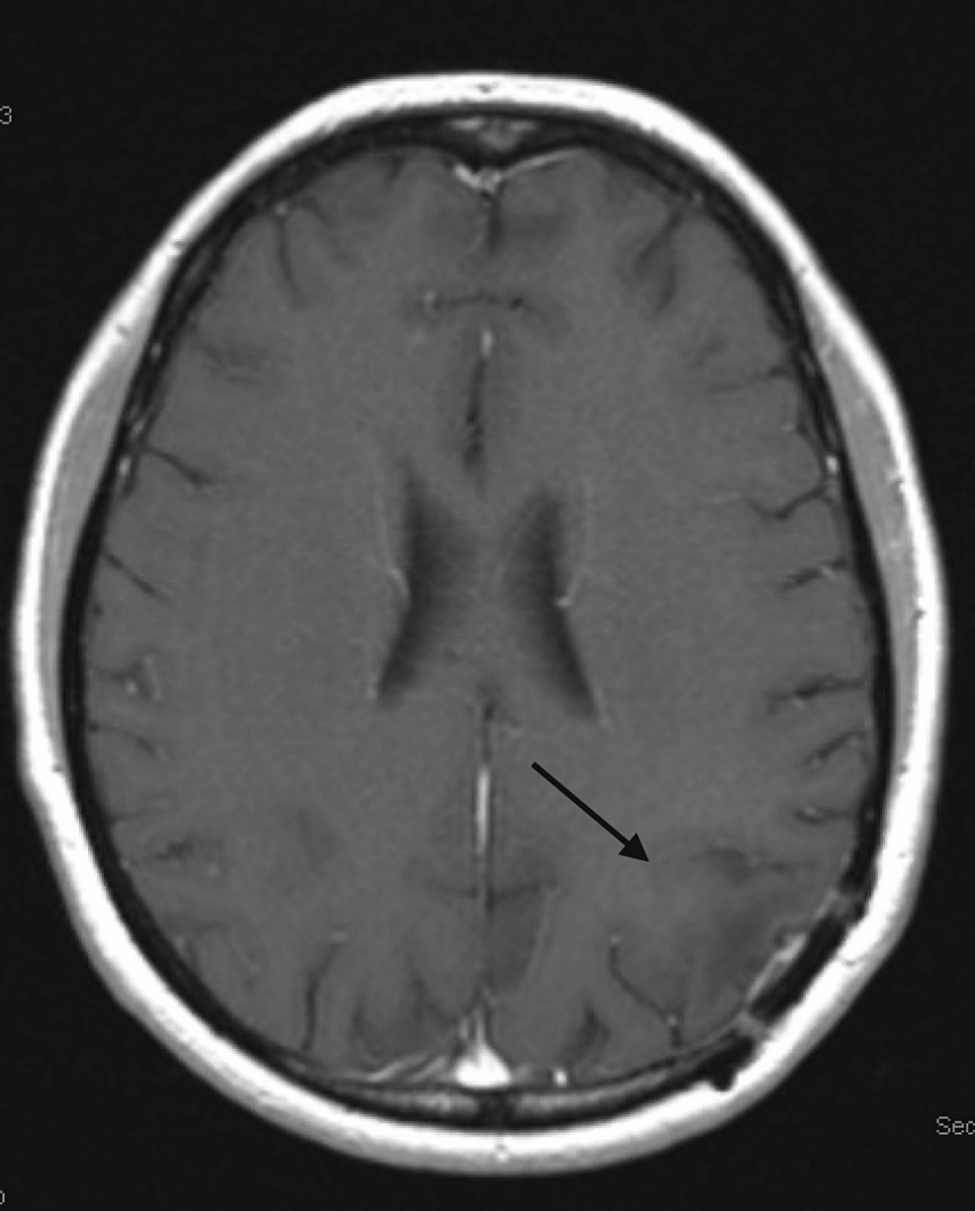
MRI of the brain showing improvement in the previously described enhancement of the resection cavity. MRI: magnetic resonance imaging

She underwent resection of the lesion by neurosurgery. Pathology showed monomorphic, CD-20 positive, EBV positive, and PTLD consistent with diffuse large B-cell lymphoma. Computed tomography of the chest, abdomen, and pelvis, MRI of the whole spine, as well as positron-emission tomography scan were negative for metastatic disease. The patient had worsening vision and she was seen by ophthalmology to evaluate the extent of the disease, visual field exam was remarkable for the right inferior quadrantanopia that correlates to previous surgery. EBV DNA titer was < 500 IU/ml in the serum. Immunosuppression including tacrolimus and mycophenolate mofetil was discontinued and she was maintained on prednisone 15 mg daily. Routine testing prior to initiating rituximab revealed chronic hepatitis B infection; therefore, the patient was started on tenofovir. She was started on rituximab for four weekly treatments at a dose of 375 mg/m^2^ with a plan to initiate high-dose methotrexate (HD-MTX) if there was less than complete response; the hesitation to initiate MTX was due to concerns regarding the patient’s fragile transplant status. MRI of the brain following the last cycle of rituximab demonstrated the progression of the disease (Figure 2). Subsequently, she received one cycle of a combination of HD-MTX 1 g/m^2^ and rituximab. The patient tolerated HD-MTX and did not have evidence of renal toxicity in laboratory studies. Therefore, she was started on a reduced dose of HD-MTX at 2 g/m^2^ every two weeks instead of the higher MTX dose range of 3.5 to 8 g/m^2^, which was a shared decision with the patient and nephrology after weighing the risk of kidney dysfunction with the possibility of a less than optimal response with regards to her lymphoma. She was followed by an MRI of the brain, which demonstrated a complete response after four cycles of treatment (Figure 3). Upon achieving complete resolution, further consolidation treatments with HD-MTX 2 g/m^2^ every four weeks were administered for a total of one year. The immunosuppression regimen was changed to everolimus after the first consolidation cycle. The patient has had stable disease for one year following the completion of chemotherapy. She was followed with a surveillance MRI of the brain every three months and a recent MRI continues to show no evidence of disease.

Our patient was able to maintain the graft function of her transplanted kidney and did not require any dialysis or rescue measures for her MTX clearance apart from the usual measures of fluids and leucovorin. Hepatitis B titers remained undetectable with tenofovir treatment.

## Discussion

PTLD can affect any organ transplant patient on immunosuppression, and it can occur following a solid organ transplant such as heart, lung, liver, kidney, and pancreas as well as, following a hematopoietic cell transplant [8]. The incidence of PTLD in kidney transplant recipients is lower compared to other solid organs; this could be attributed to the choice of immunosuppression regimen, which tends to be less aggressive than for other solid organ transplants [9]. Furthermore, studies have investigated the choice of immunosuppressant and its relationship to the incidence of PTLD in solid organ transplant recipients, but no clear trends have emerged to show a correlation between specific medications and the disease [4,10].

Prognosis in patients with PTLD following a kidney transplant is dependent on various factors including performance status at diagnosis, serum lactate dehydrogenase level, site of disease, age, serum creatinine, International Prognostic Index score, and stage [8,10]. Survival rates range from as high as 85% for low-risk patients to 0% for very high-risk patients at 10 years. Determination of risk is based on a scoring system constructed by a prospective study of 500 adult kidney recipient patients which were followed over the period of 10 years to estimate the occurrence of PTLD in that population, the scoring system included the following factors: age > 55 years, serum creatinine level > 133 μmol/L, elevated lactate dehydrogenase, disseminated PTLD, and monomorphic histology [10]. Survival rates more specific for CNS PTLD are challenging to find given its rare incidence. One case series of 84 cases between 1997 and 2010 reported a median overall survival of 17 months, which is significantly lower than the median overall survival of 31.5 months reported in a cohort of 107 patients between 1970 and 2003, in spite of the fact that the majority of these patients were in the pre-rituximab era [4,8]. More recent data is showing improvement with a median overall survival of four years that could be attributed to a better understanding of immunosuppression, use of rituximab, advances in radiation techniques, and improved supportive care [4,9].

The optimal therapy options have not been established for CNS PTLD. The choice of therapy is directed at the balance of achieving maximum efficacy while tailoring to the existing comorbidities of each individual patient. The main options of therapy for CNS lymphoma include reduction of immunosuppression and utilization of rituximab as monotherapy or in combination with chemotherapy and whole-brain radiation. These therapies have been used in combination and in tandem [11-13]. Our patient’s case was complicated by her history of two previous kidney transplants and stage 3 chronic kidney disease, which led to difficulties in dosing MTX in an effort to prevent potential renal toxicity whilst not compromising the efficacy of treatment. Due to the unavailability of clear guidelines, treatment was mainly guided by limited retrospective data and expert opinion.

HD-MTX is defined as any MTX dose above 500 mg/m^2^, and it has been used to treat some pediatric and adult malignancies including CNS lymphomas [14,15]. While highly effective, it can come with many potential side effects including nephrotoxicity through a variety of mechanisms, such as tubular damage due to the crystallization of the drug in the renal tubules [14]. The toxicity of HD-MTX is related to delayed excretion and plasma levels of the drug, which correlate with baseline kidney function and the dose of MTX used [14,16]. The estimated risk of having kidney injury with HD-MTX is approximately 2% [16,17]. Dose adjustment for kidney function is important to help protect kidney function as well as to avoid prolonging hospitalization and the costs associated with using expensive reversal agents such as glucarpidase, which may be needed if levels remain elevated despite usual measures and must be used earlier than 60 hours for the maximal benefit [18].

In our patient, the initial treatment was with a reduction of immunosuppression and a trial of rituximab, which did not yield a response. HD-MTX was then considered after discussion with the patient, within the constraints of potential toxicities in the setting of her chronic kidney disease and fragile transplant status after undergoing two kidney transplants. Potential toxicities from other therapies, such as brain radiation therapy, are factored into the final decision. She received one cycle of the combination of HD-MTX 1 g/m^2^ and rituximab. The patient tolerated HD-MTX and did not have evidence of renal toxicity in laboratory studies. Following that, she was started on a reduced dose of HD-MTX at 2 g/m^2^ every two weeks instead of the higher MTX dose range of 3.5 to 8 g/m^2^, which was a shared decision with the patient and nephrology after weighing the risk of kidney dysfunction with the possibility of a less than optimal response with regards to her lymphoma. MRI of the brain following four cycles of treatment showed complete resolution. The patient was able to achieve and maintain response one year following the completion of therapy and continues to do well.

## Conclusions

Our case highlights the significance of tailoring therapy choices for each individual based on their comorbidities and clinical response. It also suggests that there may be merit in exploring the use of reduced doses of HD-MTX in patients at high risk of renal toxicity due to kidney transplant status or chronic kidney disease, however, additional research is still required to identify safe and effective dose ranges of MTX for CNS PTLD.
